# Optimisation of a Microfluidic Method for the Delivery of a Small Peptide

**DOI:** 10.3390/pharmaceutics13091505

**Published:** 2021-09-18

**Authors:** Felicity Y. Han, Weizhi Xu, Vinod Kumar, Cedric S. Cui, Xaria Li, Xingyu Jiang, Trent M. Woodruff, Andrew K. Whittaker, Maree T. Smith

**Affiliations:** 1 Faculty of Medicine, School of Biomedical Sciences, The University of Queensland, Brisbane, QLD 4072, Australia; weizhi.xu@uq.net.au (W.X.); v.kumar1@uq.edu.au (V.K.); s.cui@uq.edu.au (C.S.C.); xaria.li@uq.edu.au (X.L.); t.woodruff@uq.edu.au (T.M.W.); maree.smith@uq.edu.au (M.T.S.); 2 Australian Institute for Bioengineering and Nanotechnology, The University of Queensland, Brisbane, QLD 4072, Australia; a.whittaker@uq.edu.au; 3 National Center for Nanoscience and Technology, Beijing 100190, China; xingyujiang@nanoctr.cn; 4 Department of Biomedical Engineering, Southern University of Science and Technology, Shenzhen 518055, China; 5 ARC Centre of Excellence in Convergent Bio Nano Science and Technology, The University of Queensland, Brisbane, QLD 4072, Australia

**Keywords:** drug delivery system, nanoparticles, poly (lactic-*co*-glycolic acid) (PLGA), microfluidic, pharmacokinetics (PK) and biodistribution

## Abstract

Peptides hold promise as therapeutics, as they have high bioactivity and specificity, good aqueous solubility, and low toxicity. However, they typically suffer from short circulation half-lives in the body. To address this issue, here, we have developed a method for encapsulation of an innate-immune targeted hexapeptide into nanoparticles using safe non-toxic FDA-approved materials. Peptide-loaded nanoparticles were formulated using a two-stage microfluidic chip. Microfluidic-related factors (i.e., flow rate, organic solvent, theoretical drug loading, PLGA type, and concentration) that may potentially influence the nanoparticle properties were systematically investigated using dynamic light scattering and transmission electron microscopy. The pharmacokinetic (PK) profile and biodistribution of the optimised nanoparticles were assessed in mice. Peptide-loaded lipid shell-PLGA core nanoparticles with designated size (~400 nm) and a sustained in vitro release profile were further characterized in vivo. In the form of nanoparticles, the elimination half-life of the encapsulated peptide was extended significantly compared with the peptide alone and resulted in a much higher distribution into the lung. These novel nanoparticles with lipid shells have considerable potential for increasing the circulation half-life and improving the biodistribution of therapeutic peptides to improve their clinical utility, including peptides aimed at treating lung-related diseases.

## 1. Introduction

Polymeric nanoparticles have been widely studied for decades as a strategy to produce sustained release of encapsulated drugs as well as enhanced therapeutic effects [1,2]. Poly (lactic-*co*-glycolic acid) (PLGA) is superior to many other polymers used for fabricating nanoparticles due to its biodegradability and biocompatibility [3]. Importantly, PLGA is approved for clinical applications involving systemic dosing routes by many international regulatory agencies including the United States Food and Drug Administration (FDA) [4].

In the last two decades, considerable effort has been directed at improving formulation methods for producing polymeric nanoparticles. Versatile methods used to fabricate PLGA nanoparticles include nanoprecipitation, emulsion and hydrogel template methods, the use of supercritical CO_2_, spray drying, coacervation, microfluidics, and the PRINT technique (particle in non-wetting templates) [5,6,7,8,9]. Compared with other methods, the microfluidic method has significant advantages, including the precise control over particle parameters (e.g., size, morphology, and charge) [10,11], monodispersed particles, automated and single-step formulation, as well as relatively high encapsulation efficiency [12,13,14]. This relatively novel formulation method provides homogeneous mixing conditions by precisely controlling micro flows, which cannot be achieved in conventional bulk methods [15,16]. Using microfluidics, the physicochemical properties of nanoparticles, including size, shape, structure, rigidity, and surface modification, can be controlled [16,17]. Additionally, lipid shell coated PLGA nanoparticles fabricated by a microfluidic method further extended the circulation half-life [18] and enhanced tumour accumulation and therapeutic effect [19].

Particle size and distribution are key parameters of nanoparticle-based drug delivery systems, because they affect multiple parameters including the blood circulation half-life [20,21], cellular uptake [21] and tumour penetration [22,23], toxicity, in vivo targeting, and metabolism [1,21]. The particle size needs to be optimized for different biomedical applications [22,24,25]. A recent study using a microfluidic method reported that PLGA particle size influences the drug release characteristics, cellular uptake, and in vivo clearance of the particles thus produced [10]. Our two-stage polydimethylsiloxane (PDMS) microfluidic chip was developed for the production of lipid-polymeric nanoparticles with varying water content to regulate the rigidity of nanoparticles [26]. This microfluidic chip had a maximum flow rate of up to 410 mL/h, enabling high-throughput synthesis of PLGA nanoparticles [27]. Additionally, this microfluidic chip can generate PLGA nanoparticles of various sizes by changing the flow rate and the flow rate ratio in the channel. We have also shown that increasing the total flow rate from 41 mL/h to 246 mL/h in the microfluidic method tends to form smaller particles [15]. However, particle size will also be influenced by a range of other factors, including drug loading, polymer chemistry, polymer concentration, additives, and other formulation conditions [6,28]. The innovation or optimization of microfluidic systems will be facilitated with the development of advanced manufacturing techniques [29,30,31]. Hence, it is important to systematically investigate the chemical and physical parameters that influence particle size using the microfluidic method.

The hexapeptide, phenylpropanoic acid-[ornithine-proline-d-cyclohexylalanine-tryptophan-arginine] (HC-[OPdChaWR]) is an antagonist of the C5a receptor 1 (C5aR1) of the innate immune complement cascade [32,33]. It has been shown to have efficacy in several animal disease models, such as inflammatory bowel disease, amyotrophic lateral sclerosis, allergic asthma, and spinal cord injury [34,35,36,37]. Typical of most peptides, however, it has a short plasma half-life in vivo (i.e., 0.17 h in mice following intravenous (IV) injection) and low oral bioavailability [38,39,40]. Utilising a nanoparticle drug delivery system has the potential to extend the circulation half-life of peptides, such as the C5aR1 antagonist investigated herein.

In this study, C5aR1 antagonist peptide-loaded lipid shell-PLGA core nanoparticles were fabricated using our two-stage microfluidic chip, and various formulation parameters were optimized to give the desired particle size distribution as well as a sustained in vitro release profile. Parameters in the microfluidic method that have the potential to influence particle size distribution were investigated. These included microfluidic flow rate, type of organic solvent utilized, theoretical drug loading, as well as PLGA types and concentration. The best performing nanoparticle formulations were identified for in vitro release investigation. The optimised peptide-loaded lipid shell-PLGA core nanoparticles were further characterized for drug incorporation efficiency and morphology. The in vivo pharmacokinetics (PK) and biodistribution of the optimised nanoparticle formulation were also assessed in mice.

## 2. Materials and Methods

### 2.1. Materials

The C5aR1 antagonist peptide (HC-[OPdChaWR]) was synthesised as previously described [41]. Poly (lactic-*co*-glycolic acid) (PLGA) (50:50, acid terminated, MW 24,000~38,000 Da) was purchased from Sigma–Aldrich Pty Ltd. (Sydney, NSW, Australia). Methoxy poly (ethylene glycol)-*b*-poly (lactide-*co*-glycolide) MW ~5000:55,000 Da (mPEG5K-PLGA55K) was purchased from PolySciTech (Akina, West Lafayette, IN, USA). Additionally, 1,2-Dihexadecanoyl-*sn*-glycero-3-phosphocholine (DPPC), 1,2-distearoylsn-glycero-3-phosphoethanolamine-N-[methoxy (polyethylene glycol (DSPE-PEG) and cholesterol were obtained from Avanti Pty Ltd. (Tonawanda, NY, USA). Phosphate-buffered saline (PBS) tablets were from Astral Scientific (Sydney, NSW, Australia). Dichloromethane (DCM) and ethanol (EtOH) were from Biolab Pty Ltd. (Melbourne, VIC, Australia). Acetonitrile (ACN) was from Ajax Finechem Pty Ltd. (Brisbane, QLD, Australia). Methanol (MeOH) was bought from RCI Labscan Ltd. (Sydney, NSW, Australia). Ethyl acetate (EA), trifluoroethanol (TFE), and dimethylformamide (DMF) were from Sigma Aldrich (Sydney, NSW, Australia). All solvents used in the study were of analytical and/or high-performance liquid chromatography (HPLC) grade. Zolazepam and xylazine were obtained from Provet, Brisbane, QLD, Australia.

### 2.2. Preparation of Peptide-Loaded Nanoparticles

**Figure 1:**
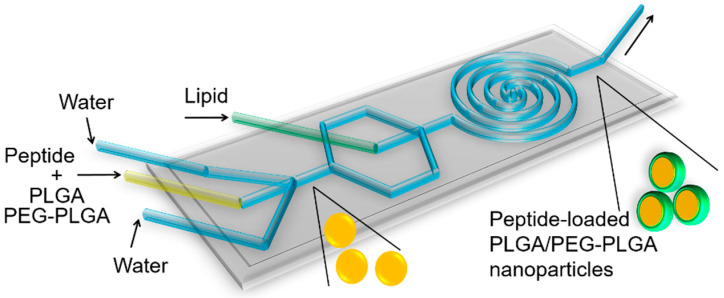
Schematic illustration of the microfluidic chip and the nanoparticle formulation process. There are four inlets and one outlet in the microfluidic chip. Peptide-PLGA solution is injected through the middle inlet while water is injected through two side inlets. The peptide-loaded PLGA nanoparticles were formed at this stage. Lipid injected through the central inlet forms the lipid shell structure of the PLGA nanoparticles. Fabricated peptide-loaded lipid shell-polymer core nanoparticles are collected from the outlet.

Peptide-loaded nanoparticles were fabricated using an optimised microfluidic method [42,43]. The two-stage microfluidic chip (Figure 1) was designed and fabricated [15] in the laboratory of Professor Xingyu Jiang. Briefly, the microfluidic chip was fabricated on a SU8-2100 master mould based on standard soft lithography. The two-stage microfluidic chip was composed of a first stage (three inlets and a straight mixing channel, 100 µm in width and 60 µm in height) and a second stage (a centre inlet and a mixing spiral channel, 300 µm in width and 60 µm in height). To obtain an appropriate ratio of thickness to height of the channels (60 μm), SU8-2100 photoresist (MicroChem Corp, Westborough, MA, USA) was spin-coated on a 4 inch silicon wafer at 500 rpm for 10 s and then at 3400 rpm for 60 s. The spin-coated SU8-2100 photoresist was baked at 65 °C for 5 min and at 95 °C for 10 min. The spin-coated SU8-2100 photoresist was exposed to ultraviolet light (150 mJ·cm^−2^) through a photomask containing the pattern of channels. The exposed photoresist was baked at 60 °C for 6 min and 110 °C for 8 min. SU-8 developer (MicroChem Corp) was used to dissolve the unexposed photoresist. The method for fabricating the microfluidic chip with polydimethylsiloxane (PDMS) (Sylgard 184, Dow Corning Inc., Midland, MI, USA) and the master mould was the same as the previous work [27]. A peptide–polymer solution was prepared by dissolving peptide and PLGA in an organic solvent. The lipid solution was DPPC (4.55 mg/mL), DSPE-PEG (0.85 mg/mL), and cholesterol (0.48 mg/mL) in ethanol. The peptide–polymer solution, lipid solution, and water were injected into the microfluidic chip. The peptide–polymer solution and lipid solution were injected through the middle inlet and central inlet with the same flow rate, respectively. Water was injected from two side inlets with the same flow rate. The flow rate for each flow inlet was precisely controlled by syringe pumps (PHD ULTRA CP, SDR Scientific Pty Ltd., Sydney, NSW, Australia). The mixture containing peptide-loaded nanoparticles was collected from the outlet side of the microfluidic chip.

To remove the organic solvent, fabricated peptide-loaded nanoparticles were stirred on ice (200 rpm) for 1 h and then placed under vacuum for 30 min using a rotary evaporator (Rotavapor R-210, BUCHI Ltd., Newmarket, UK) at room temperature (~25 °C). Peptide-loaded nanoparticles were washed twice with Milli-Q water, and centrifuged at 20,000× *g* (Avanti JE, Beckman Coulter, Indianapolis, CA, USA) for 15 min. Particles were then resuspended in a small volume of PBS or 5% glucose for subsequent experiments.

Particle size and peptide release profiles were optimised by investigating a range of organic solvents (DCM, EA, TEF + DMF (3:7, *v*/*v*) [15], ACN or acetone), multiple sides (mL/h)/centre (mL/h) flow rate ratios (sides/centre, 60/4.5 [43], 120/3 [15] and 120/1), several theoretical drug loadings (5, 10, and 20%), as well as various PLGAs (PLGA or PLGA + PEG-PLGA) and polymer concentrations (1 or 2%). The flow rates of the two side (water) inlets were controlled at 60 mL/h, while the centre (polymer + drug) inlet flow rate was 4.5 mL/h. When the flow rates of the two side (water) inlets were increased to 120 mL/h, the centre inlet flow rate was reduced to either 3 or 1 mL/h. Detailed information on the parameters for all formulations prepared is summarised in Supplementary Table S1. The flow rates were specified for various total flow rates and flow rate ratios (water in the side inlets to solvent in the centre inlet) calculated using the equations listed in Supplementary Table S2.

### 2.3. Particle Size, Polydispersity Index (PDI) and Zeta Potential

The size, size distribution, PDI, and zeta-potential of the nanoparticles were determined using dynamic light scattering (DLS) at a scattering angle of 173° (Zetasizer Nano ZS, Malvern Instruments, Worcestershire, UK).

### 2.4. Incorporation Efficiency

The peptide-loaded nanoparticle suspension (200 μL) was freeze-dried (*n* = 3) and then completely dissolved in 1 mL of acetonitrile, and the peptide concentration was quantified using LC-MS/MS [38]. The actual drug loading (%, *w*/*w*) and the drug incorporation efficiency, expressed as encapsulation efficiency (EE, %, *w*/*w*) were calculated using the equations below. The individual values for three replicate determinations and their mean values are reported.

Actual drug loading (%) = w(drug)/[w(drug) + w(polymer)](1)

EE (%) = w(drug in particles)/w(drug added)(2)

### 2.5. In Vitro Release Profile

The peptide-loaded nanoparticle suspension (200 μL) was transferred into dialysis tubes (MWCO 10 kDa). Each dialysis tube was sealed, placed into a capped container containing 5 mL PBS, and then placed into an incubator maintained at 37 °C and shaken horizontally at 100 shakes min^−1^ (IKA^®^ KS130 shaker; Sigma–Aldrich Pty Ltd., Sydney, NSW, Australia). On each testing occasion (i.e., 1, 3, 6, 24, 30, 48, 54, and 72 h), the dialysis tubes were rinsed with 1 mL of PBS and transferred to 5 mL of fresh PBS. Concentrations of HC-[OPdChaWR] were determined in triplicate by LC-MS/MS [38] at each time of assessment, and the data are presented.

### 2.6. Measurement of Peptide by Liquid Chromatography–Tandem Mass Spectrometry (LC–MS/MS)

The C5aR1 peptide antagonist concentrations were quantified by a previously developed and validated LC–MS/MS method [38]. Briefly, the LC–MS/MS system consisted of an API 3200 (AB SCIEX) triple quadrupole LC–MS/MS mass spectrometer coupled with an Agilent 1200 series HPLC system (Agilent Technologies, Santa Clara CA, USA). For sample processing, 10 µL of the solution containing the C5aR1 peptide antagonist was mixed with 10 µL of internal standard (AcF-[OPdChaWR]) dissolved in solvent (ACN) followed by sample processing, extraction, supernatant isolation, drying, and reconstitution in a 75% methanol-in-water solution. A small volume of processed sample (approximately 50 µL) was used for the LC–MS measurement. The autosampler was set at 4 °C and the column oven temperature was maintained in the range of 25 ± 1 °C. The system control and data acquisition were executed by Analyst software (AB SCIEX, Applied Biosystems Inc., Framingham, MA, USA, version 1.5.1). Chromatographic separation was implemented using a Kinetex EVO C18 analytical column (100 mm × 2.1 mm, 100 Å, 5 µm, Phenomenex Inc., Torrance, CA, USA) under binary gradient conditions using mobile phase A (milliQ water containing 0.1% formic acid) and mobile phase B (acetonitrile containing 0.1% formic acid) with a variable flow rate, as described in Kumar et al. [38].

### 2.7. Morphology

The morphology of nanoparticles was observed under a transmission electron microscope (TEM, Hitachi HT7700, Hitachi High-Technologies Corporation, Tokyo, Japan) at 100 kV. Briefly, 10 µL of diluted nanoparticle suspension (1 mg/mL) was dropped onto a carbon-coated copper grid and incubated for 10 min to allow nanoparticles to attach to the membrane. Next, samples were negatively stained with 1% uranyl acetate and viewed.

### 2.8. Pharmacokinetics Study and Biodistribution

All experimental procedures involving animals were performed following approval from the animal ethics committee of the University of Queensland (The approval number is 241/18, and the date of approval is 21 August 2018). All experiments were conducted as per the National Health and Medical Research Council of Australia policies and guidelines for the care and use of animals for scientific purposes (8th Edition, 2013). Wild-type C57BL/6J mice (male, 10–12 weeks old) were purchased from the Animal Resources Centre (Perth, WA, Australia). All animals were housed within the University of Queensland Biological Resources animal facility in a pathogen-free environment with a 12 h dark/12 h light cycle and free access to food and water.

The plasma pharmacokinetic (PK) profile and biodistribution of peptide-loaded nanoparticles (1 mg/kg equivalent dose of free peptide) vs. free peptide were assessed in mice after intravenous (IV; via tail vein) dosing. Serial blood samples were collected via a tail vein using a microsampling technique [40]. Blood samples were collected at 0.04, 0.25, 0.5, 0.75, 1, 1.5, 1, 4, 6, and 24 h for both groups and then 48, 72, 96, and 120 h for the nanoparticle group. The blood samples were centrifuged at 2000× *g* for 10 min at 4 °C, and the separated plasma samples (10 µL) were collected, diluted with 100 µL of milliQ water and 10 µL of internal standard, filtered through Microcon^®^-10 centrifugal filters (Merck, Melbourne, VIC, Australia) to separate nanoparticles from released peptide, and stored at −80 °C until time of analysis. For tissue distribution, on the terminal day, i.e., day 1 for native peptide and day 5 for the nanoparticle group, animals were transcardially perfused using PBS solution to remove circulatory peptide/formulation, and tissue samples were collected, processed as described in our previously published method [38], and filtered as mentioned above.

On the day of analysis, samples were processed for C5aR1 peptide antagonist concentrations using the above-mentioned LC–MS/MS method as described previously [38]. The IV route of drug administration was used for this study to achieve 100% bioavailability of peptide or peptide-loaded nanoparticles in the systemic circulation of the mice.

### 2.9. Data Analysis

The data are presented as mean (±SEM) unless otherwise specified, and plasma concentration versus time curves were prepared using GraphPad Prism (GraphPad Software Inc., La Jolla, CA, USA, version 7). The statistical significance criterion was *p* ≤ 0.05. LC–MS/MS data processing and analysis were performed using Analyst software (AB SCIEX, Applied Biosystems Inc., Framingham, MA, USA, version 1.5.1) and Multiquant software (AB SCIEX, USA, version 2.0).

PK parameters were derived from the plasma concentration versus time profiles using a two-compartmental model as implemented in the WinNonlin software package (Certara, L.P, St. Louis, MO, USA, v8.1). The linear trapezoidal rule was applied for the calculation of area under the curve (AUC _0–t_) of the plasma concentration vs. time profiles.

## 3. Results and Discussion

### 3.1. Optimisation of Particle Size

#### 3.1.1. Effect of Flow Rate (Side/Centre)

**Figure 2:**
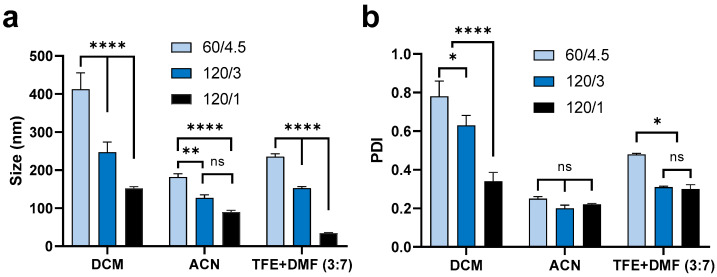
Mean (±SEM) (*n* = 3) size (**a**) and PDI (**b**) of ‘blank’ PLGA nanoparticles formulated using 1% PLGA in DCM, ACN, and TFE + DMF (3:7, *v*/*v*) under a range of side/centre flow rate conditions (water inside inlets to solvent in centre inlet, 60/4.5 = 60 to 4.5 mL/h, 120/3 = 120 to 3 mL/h, 120/1 = 120 to 1 mL/h). Using the same solvent, increasing flow rate and flow rate ratios significantly decreased the size of nanoparticles. Increasing flow rate or flow rate ratio caused a dramatic decrease in the PDI of nanoparticles formulated using DCM but did not significantly affect nanoparticles formulated using ACN or TFE + DMF (3:7). Two-way ANOVA analysis followed by Tukey test for multiple comparisons (**** *p* < 0.0001; ** *p* < 0.01; * *p* < 0.05; and ns, no significant difference).

Changing the side (mL/h)/centre (mL/h) flow rate from 60/4.5 to 120/3 then to 120/1 showed similar effects on the mean size of nanoparticles fabricated by the various organic solvents assessed (Figure 2). The mean size and PDI of particles for all formulations are summarised in Table S1. By increasing the total flow rate and flow rate ratio (water in side inlets to solvent in centre inlet) (Table S2), the size of nanoparticles fabricated using DCM decreased from ~400 to ~150 nm, the size of nanoparticles using ACN decreased from ~200 to <100 nm, and the size of nanoparticles using TFE + DMF (3:7, *v*/*v*) decreased from 250 to ~30 nm. It is worth noting that increasing the sides/centre flow rate ratio had a more significant effect on the PDI of nanoparticles formulated with DCM, resulting in a sharply decreased PDI, from 0.8 to approximately 0.3. In comparison, using a higher sides/centre flow rate ratio only caused a small effect on the PDI of nanoparticles from the TFE + DMF (3:7, *v*/*v*) formulation and no significant effect on nanoparticles from the ACN formulation. Generally, a higher total flow rate and higher flow rate ratio (water to solvent) are believed to provide a higher shear stress, which could produce smaller organic droplets and subsequently form smaller nanoparticles through precipitation [44]. Subsequently, the higher side/centre flow rate ratio results in smaller nanoparticles with greater size homogeneity (lower PDI) [15,42]. Additionally, the higher side/centre flow rate ratio results in more effective and rapid interfacial deposition, thus generating nanoparticles with more uniform size [15,42].

#### 3.1.2. Effect of Organic Solvent

**Figure 3:**
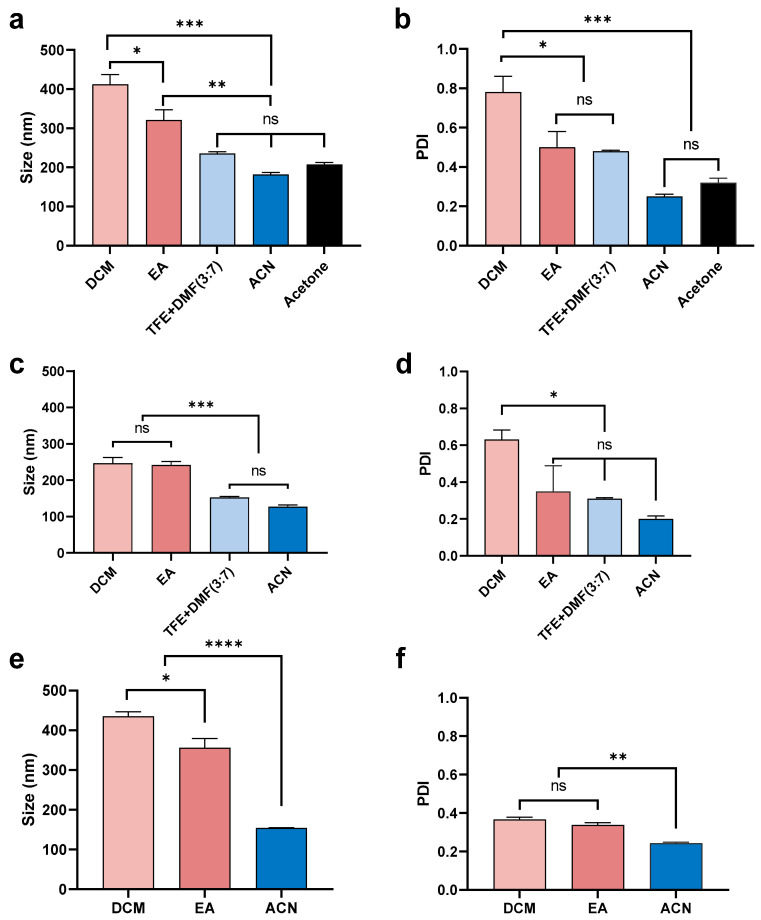
Mean (±SEM) (*n* = 3) size (**a**) and PDI (**b**) of blank nanoparticles formulated using a low sides/centre flow rate ratio (60/4.5) using 1% PLGA in a range of organic solvents (DCM, EA, TFE + DMF (3:7, *v*/*v*), ACN, and acetone). Nanoparticles fabricated using DCM had the largest size (400 nm) and the highest PDI (0.8). By comparison, use of ACN resulted in particles of the smallest size (<200 nm) and lowest PDI (~0.2). Mean (±SEM) (*n* = 3) size (**c**) and PDI (**d**) of blank nanoparticles formulated using a high sides/centre flow rate ratio (120/3) using 1% PLGA in different organic solvents (DCM, EA, TFE + DMF (3:7, *v*/*v*), and ACN). Both DCM and EA produced particles of a larger size (~250 nm) compared with ACN (~100 nm). DCM provided the highest PDI (0.6) while ACN led to the lowest PDI (<0.2). Mean (±SEM) (*n* = 3) size (**e**) and PDI (**f**) of 5% theoretical drug-loaded nanoparticles formulated with a low side/centre flow rate ratio (60/4.5) using 1% PLGA in different organic solvents (DCM, EA, and ACN). DCM provided a larger particle size (>400 nm) compared with EA (~350 nm) and ACN (150 nm). Solvents did not show a significant difference in PDI (0.2~0.3). One-way ANOVA analysis followed by Tukey test for multiple comparisons (**** *p* < 0.0001; *** *p* < 0.001; ** *p* < 0.01; * *p* < 0.05; and ns, no significant difference).

As can be seen in Figure 2, the organic solvent utilized had a significant influence on the size of nanoparticles, even if all other factors were the same. Therefore, the mean size and PDI of peptide-loaded nanoparticles (5% theoretical loading) were compared between different organic solvents. As shown in Figure 3, using ACN resulted in the smallest size (200 nm) and PDI (~0.2) under all three different side/centre flow rate ratios. The nanoparticles fabricated under a low side/centre flow rate ratio (60/4.5) using acetone and the combined solvent, TFE + DMF (3:7, *v*/*v*) had a slightly larger size (200–250 nm) and relatively high PDI (0.5 and 0.3, respectively). On the other hand, nanoparticles fabricated using DCM were the largest size (~400 nm) and had the broadest size distribution (PDI 0.8) followed by nanoparticles fabricated using EA (size 300 nm, PDI 0.5). The same trend was observed under high flow rate conditions (side/centre at 120/3), but the difference between solvents was attenuated (Figure 3c,d). Peptide-loaded nanoparticles performed similarly to the ‘blank’ nanoparticles (Figure 3e,f), such that particle sizes were 400, 350, and 150 nm, both with and without peptide for nanoparticles formulated using DCM, EA, and ACN, respectively. However, in comparison to the blank (without peptide) nanoparticles (PDI 0.3–0.8), the PDI of nanoparticles containing encapsulated peptide was decreased (PDI 0.2–0.3).

In brief, ACN and TFE + DMF (3:7, *v*/*v*) always led to the fabrication of smaller sized particles, characterized by a narrower size distribution (lower PDI), whereas DCM and EA resulted in larger particle sizes and a broader size distribution (higher PDI). This fact could be explained by the differential miscibility of the various organic solvents in water [45]. DCM and EA are water-immiscible solvents, whereas TEF, DMF, ACN, and acetone are miscible with water. In agreement with others, larger particles were fabricated using DCM compared with partially/fully water-miscible solvents [45]. Similarly, increasing the ratio of DCM in a mixed solvent resulted in increased particle size and PDI [46]. Concerning the geometry of the microfluidic chip, the PLGA dissolved in a water-miscible solvent generated a more efficient shearing force that was beneficial for self-assembly via nanoprecipitation subsequently resulting in smaller and mono-dispersed nanoparticles [47]. Additionally, liquid viscosity, density, and surface tension also have a dynamic effect on the droplet formation and particle size distribution [47,48]. However, it was noticed that the microfluidic chip was blocked easily when using ACN as the organic solvent, and this caused substantial drug loss, so this method is not feasible for producing particles in a large quantity. Additionally, the PDI of nanoparticles fabricated using DCM was smaller when the peptide was encapsulated in comparison with ‘blank’ nanoparticles (Figure 3d,f). For further investigation, a range of drug loadings were assessed for their impact on particle size distribution. DCM was used for further formulation development, as its lower boiling point means that it can be fully removed under ambient temperature and pressure. Another consideration was that our peptide is soluble in water (1 mg/mL), and so water miscible solvents are unsuitable for this purpose.

#### 3.1.3. Effect of Theoretical Drug Loading

**Figure 4:**
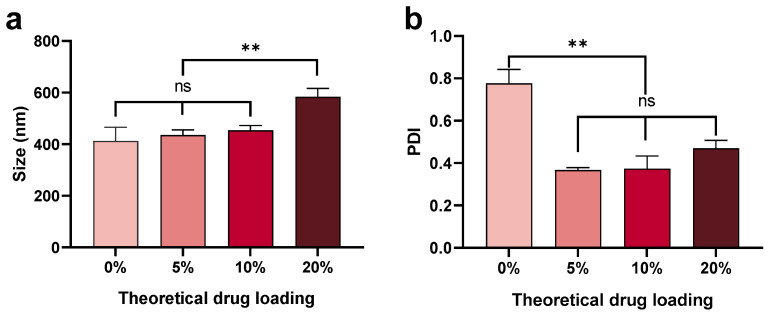
Mean (±SEM) (*n* = 3) size (**a**) and PDI (**b**) of nanoparticles with a range of theoretical drug loadings (0, 5, 10, and 20%) fabricated using 1% PLGA in DCM using a low side/centre flow rate ratio (60/4.5). The mean size of nanoparticles increased with increased drug loading, with this effect most marked at 20% peptide loading (0 < 5 < 10 < 20%). The PDI was significantly decreased for particles containing 5% peptide (<0.4) compared with blank nanoparticles (0.8). PDI increased with increased drug loading (5 < 10 < 20%). One-way ANOVA analysis followed by Tukey test for multiple comparisons (** *p* < 0.01; and ns, no significant difference).

The size of nanoparticles increased with increased theoretical drug loading (from 400 nm to 600 nm) (Figure 4). However, the nanoparticles loaded with peptide had a narrower size distribution (PDI 0.35–0.45) compared with the blank nanoparticles (PDI ~0.8). The presence of peptide aided the formation of homogenous nanoparticles, although the mechanism behind this phenomenon is unclear and needs further investigation. It is known that, during formulation, nanoparticles undergo nucleation, growth, and aggregation [49]. The C5aR1 peptide antagonist contains a hydrocinnamate residue and is sufficiently lipophilic to have a positive effect on nucleation or particle growth [32,50]. It is plausible that a hydrophobic core may form more readily by adding peptide compared with particles comprised of pure PLGA when DCM is used as the solvent in the microfluidic method. Therefore, a lower PDI was observed for particles with encapsulated peptide (Figure 4b).

#### 3.1.4. Effect of Polymer

**Figure 5:**
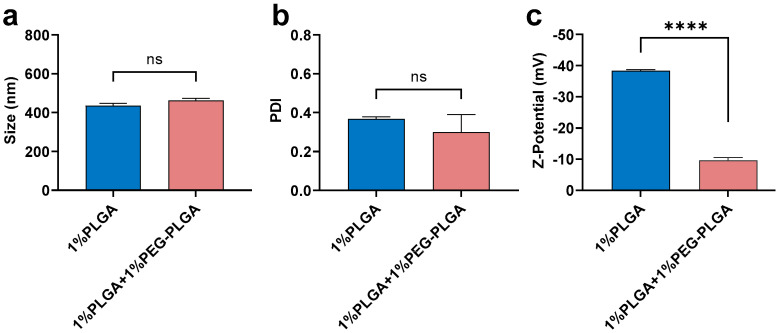
Mean (±SEM) (*n* = 3) size (**a**), PDI (**b**), and Z-potential (**c**) of peptide-loaded nanoparticles (5% theoretical drug loading) fabricated using 1% PLGA in DCM or 1% PLGA + 1% PEG-PLGA in DCM under low side/centre flow rate ratio conditions (60/4.5). The extra 1% PEG-PLGA showed a slight increment in size distribution but significantly lower Z-potential. One-way ANOVA analysis followed by Tukey test for multiple comparisons (**** *p* < 0.0001; and ns, no significant difference).

PLGA (1%) was compared with the mixture of 1%PLGA + 1%PEG-PLGA (Figure 5). There was no significant difference in size and PDI between nanoparticles fabricated using 1% PLGA or 1% PLGA + 1% PEG-PLGA when using DCM as the solvent and 5% theoretical drug loading (Figure 5a,b). This finding suggested that the addition of an extra 1% PEG-PLGA did not greatly affect particle size distribution. However, adding PEG-PLGA significantly decreased the Z-potential of nanoparticles (−10 mV) compared with PLGA alone (−40 mV) (Figure 5c). Our finding is consistent with others who have reported that PEGylation always results in a dramatic decrease in the charge of PLGA nanoparticles [51,52,53]. As reported by Garinot et al., the decreased zeta potential close to neutrality may result from the shielding effect of the PEG chains [53].

To produce drug-loaded polymeric nanoparticles, nanoprecipitation and emulsion-based methods are commonly used. Nano-precipitation is based upon the interfacial deposition of polymeric nanoparticles when water-miscible solvents containing dissolved polymer are mixed with water [54]. To generate uniform and size-controlled nanoparticles by nano-precipitation, rapid and sufficient mixing is generally required [55] and often leads to a wide range size distribution. In agreement with work by others [55,56], our data show that, using a rapid mixing microfluidic method to co-precipitate the drug and polymer, more uniform and size-controlled nanoparticles are produced.

### 3.2. Optimisation of In Vitro Release Profile and Further Characterization

**Figure 6:**
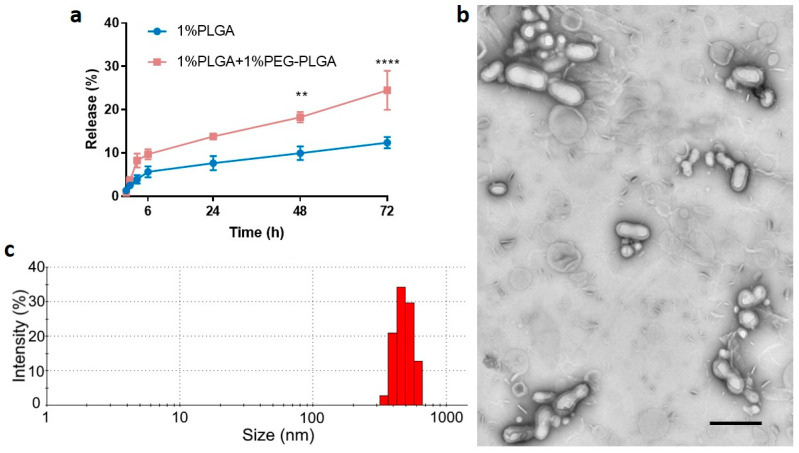
(**a**) Mean (±SEM) in vitro C5aR1 peptide antagonist release profiles (*n* = 3) for nanoparticles formulated using 1% PLGA in DCM or 1% PLGA + 1% PEG-PLGA in DCM using a low side/centre flow rate ratio (60/4.5). The percentage drug release was calculated based on actual drug loading. Two-way ANOVA analysis followed by Sidak’s test for multiple comparisons (**** *p* < 0.0001; and ** *p* < 0.01). Nanoparticles prepared using 1% PLGA + 1%PEG-PLGA in DCM were characterized for morphology (**b**) using transmission electron microscopy (TEM) and size distribution (intensity%) (**c**) using DLS. The TEM image indicates that peptide nanoparticles were successfully coated by a lipid shell as the foggy boundary could be observed. The shadowy particles in the background are components that did not coat polymer particles. The scale bar is 400 nm.

As shown in Figure 6a, peptide-loaded PLGA/PEG-PLGA nanoparticles achieved a maximum cumulative release of ~25% within 72 h, whereas the release from peptide-loaded PLGA nanoparticles was only ~13%. Additionally, PEG-PLGA was reported to form stealth nanoparticles, which have a longer in vivo circulation half-life [52]. However, more polymer concentrations and other types of PLGA with different L:G ratios, different molecular weights, and different terminal groups (e.g., ester group) remain for future investigation to achieve the optimal release profile [1].

The modified formulation of 1% PLGA + 1% PEG-PLGA in DCM and 5% theoretical drug loading using a low side/centre flow rate ratio (60/4.5) was further investigated before undertaking the in vivo study. The actual drug loading for this formulation was 2.0 ± 0.5% (mean ± SD, *n* > 3) with EE of 38.2 ± 10.6% (mean ± SD, *n* > 3). Note that the formulation with 1% PLGA alone has an actual drug loading of approximately 1.8% with the encapsulation efficiency at approximately 36%. The core–shell structure of these nanoparticles was confirmed using TEM (Figure 6b). The size distribution is shown in Figure 6c, and it is consistent with those previously determined (Figure 5). Although a few lipid components could be observed in the freshly prepared formulation, they can be readily removed through washing. The mean size of the particles was relatively large at 400 nm, consistent with previous work that showed relatively large particles had less initial burst release compared with smaller particles [57]. Additionally, particles of this size (<500 nm) are unlikely to be cleared by phagocytosis [58] before releasing their drug payload. Thus, the precise fabrication of particles at ~400 nm was achieved by using the microfluidic method and it was selected for the in vivo study.

Previously, we have reported on the release of linear and cyclic peptides from polyurethane films with different hard and soft segments. Solvent casting of the polyurethane at room temperature mixed with the various peptides resulted in reproducible efflux profiles with no evidence of drug degradation. The organic solvent used was DCM. The C5aR1 antagonist peptide retained its biological activity during casting of the polyurethane films and following release from the films [59].

### 3.3. In Vivo Pharmacokinetics Study and Bio-Distribution

**Figure 7:**
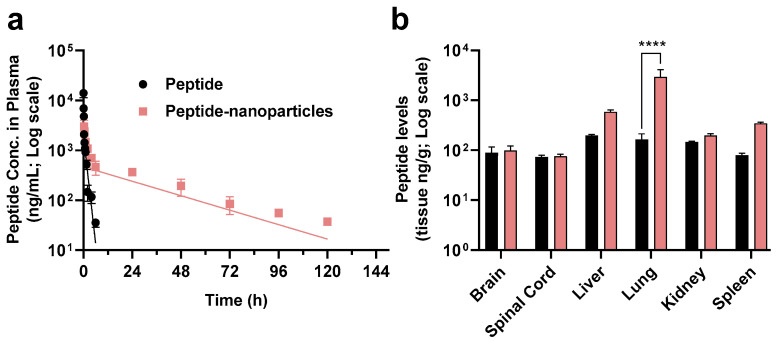
(**a**) Plasma concentration versus time profile (two-compartmental model) (mean ± SEM, *n* = 4) of the C5aR1 peptide antagonist administered by bolus intravenous (IV) injection at a dose of 1 mg/kg either as free compound (black) or in the form of lipid-shell PLGA nanoparticles (pink). (**b**) Biodistribution (mean ± SEM, *n* = 4) of peptide released from nanoparticles in different organs (brain, spinal cord, liver, lung, kidney, spleen) (ng/g). Tissues were extracted on day one and day five after a single IV injection of 1 mg/kg either as free peptide (black on day one due to the short half-life of this peptide based on our previous PK studies for the free peptide) or in the form of nanoparticles (pink on day five), respectively. Statistical analysis was conducted by two-way ANOVA, followed by Sidak’s test for multiple comparisons (**** *p* < 0.0001).

The innate immune targeted C5aR1 antagonist peptide encapsulated within the biodegradable nanoparticles had a significantly reduced elimination half-life in mice compared to the peptide itself (Figure 7a). Specifically, the plasma peptide levels after IV dosing with free peptide were unmeasurable after 6 h post-dosing whereas peptide from the nanoparticles was measurable in plasma samples for up to five days after a single IV dose administration. The terminal elimination half-life (t_1/2_) after dosing of mice with free peptide was 0.84 h, which is in agreement with the short t_1/2_ values of 0.17 h and 0.33 h reported previously [38,40]. The half-life of peptide in the form of nanoparticles was extended significantly (24.9 h). Similarly, peptide-loaded nanoparticles have a much longer mean residence time (MRT) (32.61 h) compared with that of the free peptide (0.76 h). The systemic exposure (AUC_0–t_) of peptide released from peptide-loaded nanoparticles was nearly five times larger than that for the free peptide (AUC for free peptide = 3825.7 ng.h/mL; AUC for peptide-loaded nanoparticle = 18,665.6 g.h/mL). Thus, our findings from the mouse pharmacokinetic study have confirmed that the peptide-loaded nanoparticles significantly increased the systemic exposure of this peptide when administered via the optimised nanoparticle formulation.

Additionally, the biodistribution of peptide in mice administered a single IV injection of the optimised nanoparticle formulation, showing that 18-fold higher concentrations of peptide were present in the lung tissue of the same animals for at least five days compared with animals dosed with free peptide (Figure 7b). In support of this notion, previous work by others showed that C5aR1 inhibition alleviated influenza virus-induced lung injury in mice [60]. Recently, other work showed that anti-C5a monoclonal antibody treatment improved the deteriorating health of patients with COVID-19 [61], and targeting C5aR1 with anti-C5aR1 monoclonal antibodies also has demonstrated mechanistic merit [62]. Compared with peptides, antibodies are expensive, potentially lack tissue penetrance, and some patients may also develop auto-immune reactions. Thus, alternative drug modalities that can block the activation of C5aR1 and overcome the limitations of therapeutic antibodies are urgently needed. The C5aR1 antagonist peptide-loaded lipid-shell nanoparticles described in this study may therefore have potential for more effectively treating lung inflammation, and this remains an area for future investigation.

## 4. Conclusions

In summary, our findings provide valuable information on how to precisely control the fabrication of peptide-encapsulated nanoparticles to achieve a designated size using a microfluidic device, including flow rate, organic solvent, theoretical drug loading, as well as PLGA type and concentration. Small peptide-encapsulated nanoparticles (200–400 nm) were successfully produced. This study demonstrated a marked increase in the systemic exposure and a significant increase in the terminal half-life of the same IV dose of a C5aR1 peptide antagonist administered to mice in the form of peptide-loaded nanoparticles relative to the corresponding parameters for the free peptide. We also demonstrated that the lipid-shell-PLGA core nanoparticles significantly increased the biodistribution of peptide in the lung. Further development of our method has the potential to be developed into a safe, effective delivery platform for small molecule peptide drugs in general and pave the way for their progression to clinical development.

## Supplementary Materials

The following are available online at https://www.mdpi.com/article/10.3390/pharmaceutics13091505/s1, Table S1: Formulations of peptide-loaded lipid shell-PLGA core nanoparticles, Table S2: Parameters of flow rate applied in the study.
