# Supplementary material for: Optimisation of a Microfluidic Method for the Delivery of a Small Peptide
Source: Pharmaceutics. 2021 Sep 18;13(9):1505. doi: 10.3390/pharmaceutics13091505 (PMC8468767; doi:10.3390/pharmaceutics13091505)
Supplement: Supplementary file 1 [file pharmaceutics-13-01505-s001.zip › pharmaceutics-1298519-supplementary.pdf]

pharmaceutics

Supplementary Materials: Optimisation of a Microfluidic Method for the Delivery of a Small Peptide

Felicity Y. Han, Weizhi Xu, Vinod Kumar, Cedric S. Cui, Xaria Li, Xingyu Jiang, Trent M. Woodruff, Andrew K. Whittaker, and Maree T. Smith

Table S1. Formulations of peptide-loaded lipid shell-PLGA core nanoparticles.

No.	Organic solvent	Polymer (conc.% w/v)	Drug loading (%, w/w)	Flow rate (mL/h, water/polymer)	Size (mean ± SEM)	PDI (mean ± SEM)
M1	DCM	1% PLGA	0	60/4.5	412.1 ± 43.5	0.78 ± 0.14
M2	DCM	1% PLGA	0	120/3	247.0 ± 26.9	0.63 ± 0.09
M3	DCM	1% PLGA	0	120/1	151.9 ± 4.8	0.34 ± 0.08
M4	ACN	1% PLGA	0	60/4.5	181.9 ± 8.6	0.25 ± 0.02
M5	ACN	1% PLGA	0	120/3	127.0 ± 8.1	0.20 ± 0.03
M6	ACN	1%PLGA	0	120/1	89.9 ± 4.3	0.22 ± 0.01
M7	THF+DMF (3:7)	1%PLGA	0	60/4.5	235.4 ± 7.8	0.48 ± 0.01
M8	THF+DMF (3:7)	1%PLGA	0	120/3	152.7 ± 4.1	0.31 ± 0.01
M9	THF+DMF (3:7)	1%PLGA	0	120/1	34.4 ± 1.8	0.30 ± 0.04
M10	EA	1%PLGA	0	60/4.5	321.2 ± 45.1	0.50 ± 0.14
M11	EA	1%PLGA	0	120/3	241.8 ± 16.7	0.35 ± 0.24
M12	Acetone	1% PLGA	0	60/4.5	207.9 ± 7.5	0.32 ± 0.04
M13	DCM	1% PLGA	5	60/4.5	435.3 ± 20.3	0.37 ± 0.02
M14	EA	1% PLGA	5	60/4.5	356.0 ± 40.7	0.34 ± 0.02
M15	ACN	1% PLGA	5	60/4.5	154.0 ± 1.52	0.24 ± 0.01
M16	DCM	1% PLGA	10	60/4.5	453.6 ± 18.9	0.37 ± 0.10
M17	DCM	1% PLGA	20	60/4.5	583.6 ± 32.7	0.47 ± 0.06
M18	DCM	1% PLGA + 1% PEG-PLGA	5	60/4.5	463.0 ± 17.6	0.30 ± 0.16

DCM, dichloromethane; EA, ethyl acetate; TFE, trifluoroethanol; DMF, dimethylformamide.

Table S2. Parameters of flow rate applied in the study.

Flow rate	60/4.5	120/3	120/1
Total Flow rate (mL/h)	129	246	242
Flow rate ratio	27:1	80:1	240:1

Total Flow rate (mL/h) = 2 × (Flow rate$_{water}$ + Flow rate$_{polymer}$)

Total Flow rate (mL/h) = 2 × (Flow rate$_{water}$ + Flow rate$_{polymer}$)
